# Genetic Diversity and Population Structure of *Macrobrachium nipponense* in Huaihe River Using Microsatellite Markers

**DOI:** 10.3390/genes17080880

**Published:** 2026-07-28

**Authors:** Zhiguo Hu, Yuyuan Wu, Jiahui Liu, Xusheng Guo, Baohua Duan

**Affiliations:** College of Fisheries, Xinyang Agriculture and Forestry University, Xinyang 464000, China

**Keywords:** *Macrobrachium nipponense*, microsatellite markers, genetic diversity, population structure

## Abstract

Background: The oriental river prawn (*Macrobrachium nipponense*) is an economically important aquaculture species in China. Methods: In this study, the genetic diversity and population structure of 12 populations were investigated using ten microsatellite markers. Among them, the Xiaolong Mountain (XLS) population is a cultured stock, while the Songjiachang Reservoir (SJC) population originates from the Yangtze River as an outgroup. Results: A total of 213 alleles were detected across 342 individuals, with an average of 21.3 alleles per locus. The observed heterozygosity (*Ho*) ranged from 0.362 to 0.902 (mean = 0.724), and the expected heterozygosity (*He*) ranged from 0.581 to 0.925 (mean = 0.805). Shannon’s diversity index (*I*) ranged from 1.224 to 2.810 (mean = 2.130). The polymorphism information content (*PIC*) varied from 0.532 to 0.920 (mean = 0.787), indicating high informativeness of the markers. Population structure analysis revealed a weak genetic subdivision with a subtle clustering signal (K = 2), but evaluation of LnP(K) indicated that K = 1 could not be confidently rejected. Pairwise *Fst* values ranged from 0.008 to 0.058, with high gene flow (*Nm* = 4.060–31.000), denoting low-to-moderate genetic differentiation. AMOVA showed that 98% of genetic variation resided within populations, with only 2% among populations (overall *Fst* = 0.020, *p* < 0.01). Nei’s genetic distances ranged from 0.094 to 0.277. Conclusions: Overall, the 12 populations of *M. nipponense* exhibited high genetic diversity, low-to-moderate genetic differentiation, and a weak population structure with most variation residing within populations. These findings provide valuable molecular markers for selective breeding and offer theoretical guidance for the conservation and sustainable utilization of *M. nipponense* germplasm resources, although the inferred population structure should be interpreted with caution given the weak differentiation signal.

## 1. Introduction

The oriental river prawn (*Macrobrachium nipponense*) is an important freshwater economic shrimp species in China, widely distributed in lakes, ponds, and rivers. This species is widely cultivated for its tender texture, strong reproductive ability, and short reproductive cycle [1,2]. In the past decades, *M. nipponense* production has gradually increased, reaching 223,264 tons in 2024 [3]. However, the current breeding populations generally suffer from germplasm degradation problems such as slow growth rate, uneven individual size, and weak stress resistance, which seriously restrict the high-quality development of the industry [4,5]. Genetic diversity reflects the adaptability and evolutionary potential of species in response to habitat changes. The assessment of genetic diversity is crucial for the effective management and conservation of existing genetic resources [6]. As population genetic structure determines the level and distribution of genetic diversity, investigating the genetic structure of different *M. nipponense* populations and clarifying their genetic diversity level are important bases for formulating reasonable protection strategies and fully developing germplasm resources.

Molecular markers have been widely recognized as powerful tools for investigating genetic diversity and population structure. In *M. nipponense*, most previous studies have relied on mitochondrial DNA markers, particularly the D-Loop sequences, to assess the genetic diversity and population structure of different populations. For example, Iko et al. (2025) [7] used D-loop sequences to assess genetic diversity and population structure of nine *M. nipponense* populations from saline-alkaline regions in China, revealing high diversity in four sites (Jiangsu Province (JS), Ningxia Province (NX), Jilin Province (JL), Tianjin (TJ)), close genetic relatedness between Shandong Province (SD) and Gansu Province (GS), and two main geographic clusters. Jiang et al. (2023) [8] investigated 22 wild *M. nipponense* populations using D-loop sequences, identifying 221 haplotypes and 348 polymorphic sites, and revealed high genetic diversity in southern populations as well as significant genetic differentiation among populations. In addition, a few studies have also employed the COI gene or a limited set of microsatellites (Simple Sequence Repeats, SSRs) [9,10,11]. However, mitochondrial markers are maternally inherited and show limited resolution [12]. By comparison, SSRs have been widely used in the genetic diversity analysis of aquaculture species due to their genome-wide coverage, high polymorphism, and co-dominant inheritance, offering superior resolution for detecting fine-scale population structure and gene flow [13,14,15]. Mitochondrial markers, in contrast, provide complementary information on maternal lineages and historical phylogeographic patterns [12].

The Huaihe River, as a major water system in eastern China, serves as an important habitat and germplasm resource area for *M. nipponense*, playing a critical role in the species’ distribution and genetic connectivity. Despite the ecological and economic significance of this river basin, however, there are relatively few studies on the genetic diversity and population structure of *M. nipponense* in the Huaihe River. In freshwater systems, population genetic structure is strongly influenced by hydrological connectivity, which facilitates gene flow and reduces differentiation among populations [16]. Large, interconnected river basins often exhibit weak genetic structure, high gene flow, and extensive admixture, particularly for species with high dispersal ability or when no strong physical barriers exist [17,18]. Therefore, in such systems, one may expect to find low genetic differentiation, high gene flow, and subtle clustering signals rather than discrete, well-defined subpopulations. SSR markers are sensitive to even weak differentiation; however, they are also prone to null alleles and homoplasy, and their high polymorphism can sometimes detect statistical clusters that are not biologically meaningful under high gene flow [19,20]. Consequently, results from SSR-based STRUCTURE analyses should be interpreted cautiously, and complementary metrics must be integrated to avoid overinterpreting weak signals. Therefore, this study aims to apply a set of polymorphic SSR markers to characterize the genetic diversity and population structure of *M. nipponense* across the Huaihe River, with particular attention to the degree of connectivity and the strength of differentiation. The findings are expected to provide preliminary insights for the conservation and sustainable utilization of this important fishery resource.

## 2. Materials and Methods

### 2.1. Sample Collection and DNA Extraction

**Figure 1:**
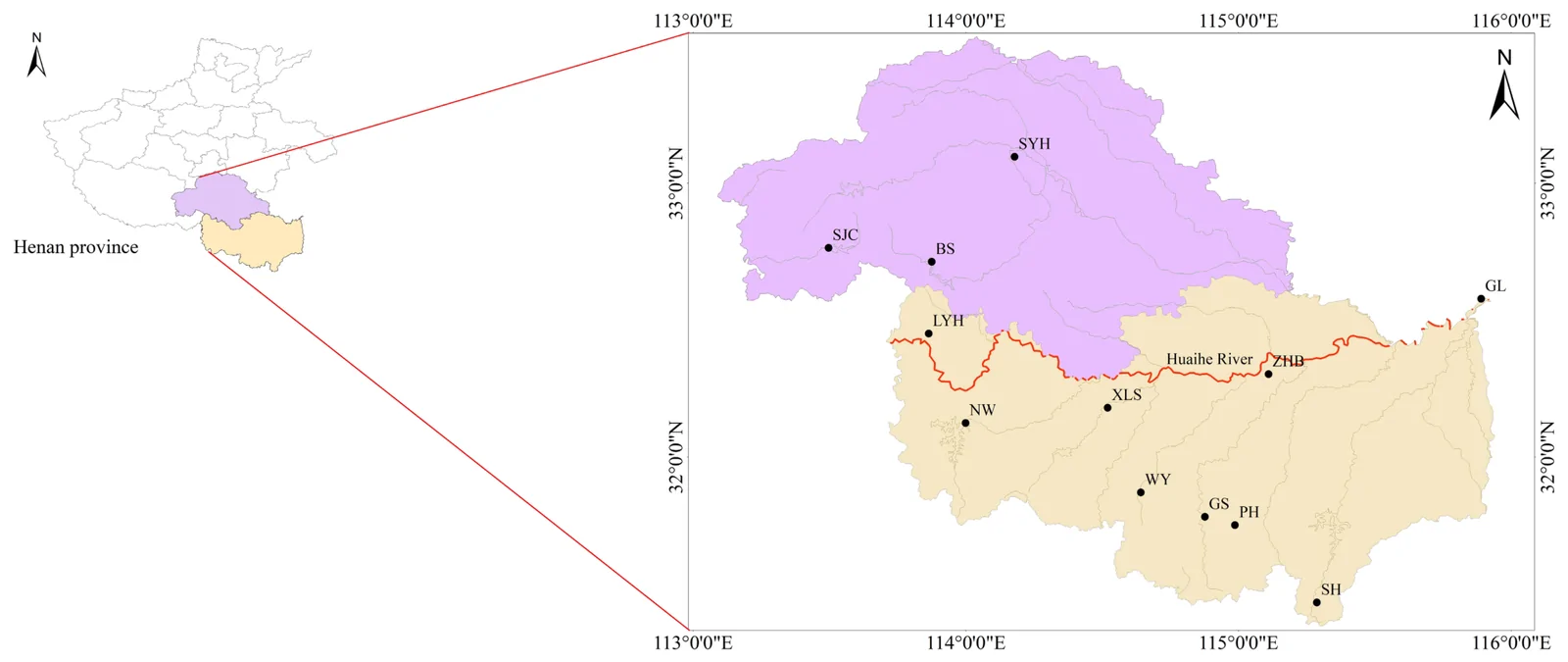
Sampling locations of twelve *M. nipponense* populations.

In this study, a total of 342 *M. nipponense* individuals were sampled from twelve sampling sites from August 2024 to January 2025 (Table 1; Figure 1). Specifically, 11 populations originated from the upper reaches of the Huai River, and another population (SJC) came from the Yangtze River. The SJC population was selected as an outgroup because the Yangtze and Huaihe River systems represent two major, historically separate drainage basins with different hydrological regimes. Including an outgroup population from a distinct river system allows us to (1) assess whether genetic differentiation between basins exceeds that within the Huaihe River, (2) calibrate the level of genetic divergence expected between geographically separated populations, and (3) evaluate whether any genetic structure detected by STRUCTURE reflects genuine population subdivision or simply the inclusion of a distantly related population. Except for the Xiaolong Mountain (XLS) population, the rest are all wild populations. Individuals were placed on ice before tissue collection to reduce activity and facilitate handling, then dissected to collect muscle tissues, which were preserved in 95% ethanol and stored at −20 °C. Genomic DNA was extracted from muscle tissue using a marine animal genomic DNA extraction kit (Tiangen, Beijing, China) according to the manufacturer’s instructions. The quantity and quality of the extracted DNA were monitored by a NanoDrop 2000 spectrophotometer (Thermo Fisher Scientific Inc., Waltham, MA, USA) and electrophoresis on a 1% agarose gel. Then, DNA samples were stored at −20 °C for subsequent experiments.

### 2.2. PCR Amplification and Fragment Analysis

The sequence of the 10 pairs of microsatellite primers is shown in Table 2. These primers were selected from previously published studies that reported high polymorphism for genetic diversity analysis of *M. nipponense* across different river systems [21,22,23,24,25]. A total of 10 μL of PCR reaction mixture was amplified using a Bio-Rad T100™ Thermal Cycler (Bio-Rad Laboratories, Inc., Hercules, CA, USA), comprising 5.0 µL 2 × Taq PCR Master Mix, 0.5 µL of each primer (10 µM stock, final concentration 0.5 µM), 1 µL template genomic DNA (~50 ng/µL, final concentration ~5 ng/µL), and 3.0 µL deionized water. PCR amplification was carried out using the following parameters: 5 min at 94 °C, followed by 35 cycles of 94 °C for 30 s, Tm for 30 s, and 72 °C for 30 s, followed by a final extension at 72 °C for 20 min. The PCR products were viewed under 1% agarose gel using a DYY-6C electrophoresis system (Beijing Liuyi Biotechnology Co., Ltd., Beijing, China). A 100 bp DNA ladder (Trans2K Plus DNA Marker, TransGen Biotech, Beijing, China) was used as the size standard. Gels were visualized and photographed under UV light using a Gel Doc™ XR + imaging system (Bio-Rad Laboratories, Inc., Hercules, CA, USA) with Image Lab™ software version 6.1.

The specificity of amplification of each SSR primer was determined by the band pattern of the PCR products, and the amplification efficiency was judged by the brightness of the PCR product bands. Subsequently, capillary electrophoresis (CE) of all SSR products was performed using an ABI 3730XL Genetic Analyzer (Applied Biosystems, Foster City, CA, USA) at Wuhan Tianyi Huayu Gene Technology Co., Ltd. (Wuhan, China). Specifically, each CE sample contained 1 μL of a tenfold dilution of PCR products, 8.5 μL Hi-Di™ formamide, and 0.5 μL GeneScan™ 500 LIZ™ size standard (Applied Biosystems). Fragment sizes were determined using an internal size standard curve generated from the LIZ500 peaks. Data were analyzed using GeneMarker^®^ v3.0.1 Fragment Analysis Software (Softgenetics LLC^®^, State College, PA, USA), and allele sizes were assigned by comparison to the internal size standard [26]. A negative control (no-template control) was included in each PCR run and confirmed by agarose gel electrophoresis; no amplification was detected. A positive control (a previously genotyped sample with a known allele profile) was not routinely included in the CE step because successful locus amplification was already verified by gel electrophoresis, and accurate sizing relied solely on the internal lane standard and the software’s standard curve. Instrument performance was validated by running the size standard and by comparing results with previously confirmed samples from the same SSR panel.

### 2.3. Genetic Diversity Analysis

To unravel the genetic diversity of all *M. nipponense* populations, several genetic parameters were calculated by GenAlEx version 6.501 [27], including the number of alleles (*Na*), effective allele number (*Ae*), observed heterozygosity (*Ho*), Nei’s expected heterozygosity (*He*), Shannon’s diversity index (*I*), Hardy–Weinberg equilibrium (*HWE*), and inbreeding coefficient (*Fis*). The null allele frequency (*Fna*) for loci was calculated using GENEPOP’007 [28]. Moreover, all *HWE* tests, *Fis* estimates, and *Fna* were calculated for each population (i.e., within each of the 12 populations) because these are inherently population-level statistics [29]. For each test, significance levels were adjusted for multiple comparisons using the Benjamini–Hochberg false discovery rate (FDR) correction, with a significance threshold of α = 0.05 after correction [30]. This approach avoids inflation of type I error when evaluating numerous population-locus pairs. Polymorphic information content (*PIC*) of each SSR locus was calculated by the PICalc function from the R package PopGenKit v1.0 [31].

### 2.4. Population Structure Analysis

Genetic distances among populations were calculated using PowerMarker v3.25 [32]. Population structure analysis was conducted on 342 samples using STRUCTURE v2.3.4 [33]. The number of clusters (K) was set from 1 to 15, with a burn-in period of 10,000 iterations followed by 500,000 Markov Chain Monte Carlo (MCMC) iterations. Ten independent runs were performed for each K value, and the optimal ΔK value (which indicates the most likely number of subpopulations) was calculated by CLUMPP [34] and Distruct v1.1 [35] based on the procedure of Evanno et al. (2005) [36]. Ultimately, a structure plot was generated based on the optimal K value. In addition, the variation and differentiation within and among populations, as well as their significance, were calculated using GenAlEx version 6.501. The genetic differentiation coefficient (*Fst*) and gene flow (*Nm*) were estimated, with Nm calculated according to the formula: *Nm* = 0.25 (1-*Fst*)/*Fst*. Significance levels for pairwise *Fst* comparisons and AMOVA components were estimated using 10,000 permutations. The Benjamini–Hochberg false discovery rate (FDR) correction was applied to all pairwise *Fst* comparisons (66 tests) and all HWE tests (120 population–locus pairs), with a significance threshold of α = 0.05 after correction [30]. In addition to the ΔK method, the most likely number of clusters was also evaluated following the original approach of Pritchard et al. (2000) [33] by examining the posterior probability LnP(K) across K = 1–15. If LnP(K) increases monotonically and plateaus at higher K values without a clear peak, this would suggest weak population structure and that K = 1 may be a more appropriate explanation. To evaluate the robustness of the main findings, a sensitivity analysis was performed by excluding locus Mni076, which showed evidence of null alleles and frequent Hardy–Weinberg deviations. Pairwise *Fst* and *Nm* were recalculated using the remaining nine loci, and the STRUCTURE analysis was repeated with K = 1–15 under the same parameters described above. Principal component analysis (PCA) was performed using the ‘prcomp’ function in R v4.3.2 based on the standardized allele frequency data. The first two principal components were plotted to visualize genetic relationships among individuals, and the percentage of variance explained by each component was calculated. PCA biplots were generated using the ‘ggplot2’ package v3.4.4.

## 3. Results

### 3.1. Polymorphism of Microsatellite Loci

A total of 213 alleles (*Na*) were detected across 342 individuals using 10 SSR primer pairs (Table 2). The number of alleles (*Na*) ranged from 11 to 32, with an average of 21.3 alleles per locus (Table 3). The total number of effective alleles (*Ae*) was 70.977, ranging from 2.384 (Mni093) to 13.354 (WXM06), with a mean of 7.098 per locus. Shannon’s information index (*I*) varied from 1.224 (Mni093) to 2.810 (WXM06) (mean = 2.130). The observed heterozygosity (*Ho*) was between 0.362 (Mni076) and 0.902 (WXM06) (mean = 0.724). The expected heterozygosity (*He*) ranged from 0.581 (Mni093) to 0.925 (WXM06), with a mean of 0.805. The polymorphism information content (*PIC*) ranged from 0.532 (Mni093) to 0.920 (WXM06), with an average of 0.787. These results indicated that all loci exhibited high polymorphism and were suitable for population genetic analysis.

Because *HWE* deviations, *Fis*, and *Fna* are population-level statistics, these parameters were examined for each of the 12 populations separately (Table 4 and Table S1). After FDR correction for multiple comparisons, significant HWE deviations were observed in 24 out of 120 (20%) population-locus pairs. The locus showing the most frequent deviations was Mni076, which deviated from *HWE* in 11 populations (11/12), followed by Mni082 (4/12). In contrast, loci Mni30 and WXM06 did not deviate from *HWE* in any population. The inbreeding coefficient Fis was positive in 73 out of 120 (60.83%) population-locus pairs, with a mean *Fis* of 0.071 across all pairs. However, significantly positive *Fis* (*HWE* deviation with *Fis* > 0 after correction) was observed in only 24 pairs (20%), primarily driven by locus Mni076 (11 pairs). This indicates that the majority of positive *Fis* values are not statistically significant and may be due to sampling variation rather than true inbreeding.

### 3.2. Genetic Diversity Within Populations

*Ae* and *He* among the 12 *M. nipponense* populations were in the ranges 4.783–6.699 (mean = 5.829) and 0.742–0.803 (mean = 0.773) (Table 4), respectively, indicating that all populations maintained relatively high levels of genetic diversity. Among them, the Guangshan Nature Reserve (GS) population exhibited the highest effective number of alleles (*Ae* = 6.699), followed by the Suyahu Reservoir (SYH) (*Ae* = 6.663) and Huai River mainstream (GL) (*Ae* = 6.358) populations, while the XLS population had the lowest (*Ae* = 4.783). SYH population showed the highest expected heterozygosity (*He* = 0.803), followed by the Pohe Reservoir (PH) (*He* = 0.796) and GS (*He* = 0.793) populations, with the XLS population having the lowest (*He* = 0.742). However, the observed heterozygosity of each group was less than the expected heterozygosity. The potential causes of this heterozygote deficiency are examined in the Discussion in relation to locus-specific effects and null alleles.

### 3.3. Genetic Variation Among Populations

The genetic differentiation index (*Fst*) and gene flow (*Nm*) among the 12 *M. nipponense* populations are shown in Table 5. Pairwise *Fst* values ranged from 0.008 to 0.058, with corresponding *Nm* values ranging from 4.060 to 31.000 (Table 5). After FDR correction for multiple comparisons (66 pairwise tests, α = 0.05), 18 out of 66 comparisons showed significant genetic differentiation (*p* < 0.05). The SJC population (Yangtze River outgroup) exhibited significant differentiation from 10 out of 11 Huaihe River populations (*Fst* = 0.039–0.058, *p* < 0.05), with the only non-significant comparison being SJC vs. PH (*Fst* = 0.039, *p* > 0.05). Among the Huaihe River populations, significant differentiation was observed in 8 out of 55 pairwise comparisons, with *Fst* values ranging from 0.011 to 0.026 (*p* < 0.05). The GL and SH populations exhibited the lowest *Fst* (0.008) and the highest *Nm* (31.000), indicating close genetic relatedness. In contrast, the LYH and SJC populations showed the highest *Fst* (0.058) and the lowest *Nm* (4.060), representing the greatest differentiation observed in this study. Overall, the majority of pairwise comparisons among Huaihe River populations were not significant, confirming weak genetic differentiation and extensive gene flow within the basin.

The genetic distances among the 12 *M. nipponense* populations ranged from 0.094 to 0.277, with an average of 0.1563, indicating an overall intraspecific level of differentiation (Table 6). Among them, the PH and Nanwan Reservoir (NW) populations exhibited the closest genetic distance (0.094), suggesting a relatively close genetic relationship. In contrast, the SJC and Boshan Reservoir (BS) populations showed the largest genetic distance (0.277), showing considerable genetic divergence. For all other pairwise comparisons, the genetic distances were less than 0.2, suggesting that genetic variation remains within the intraspecific level. Moreover, AMOVA revealed that the majority of genetic variation (98%) resided within populations, with only 2% occurring among populations. The overall fixation index (*Fst* = 0.020) was low but statistically significant (*p* < 0.01; Table 7). This result indicates that while genetic differentiation is detectable, it is very weak, and the *M. nipponense* populations from the upper reaches of the Huaihe River are essentially undifferentiated.

### 3.4. Genetic Structure Among Populations

**Figure 2:**
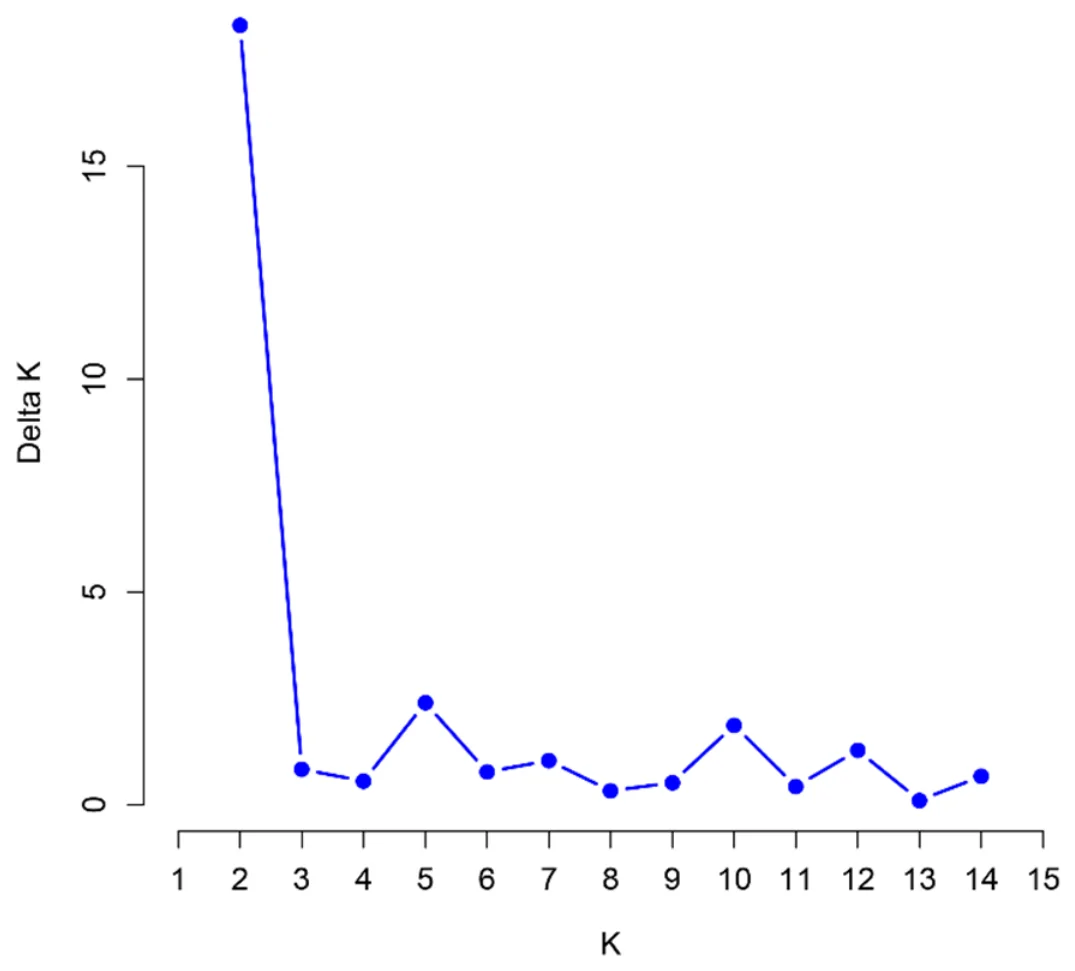
ΔK values across K = 1–15. The peak at K = 2 indicates the optimal cluster number under the Evanno et al. (2005) method [36].

**Figure 3:**
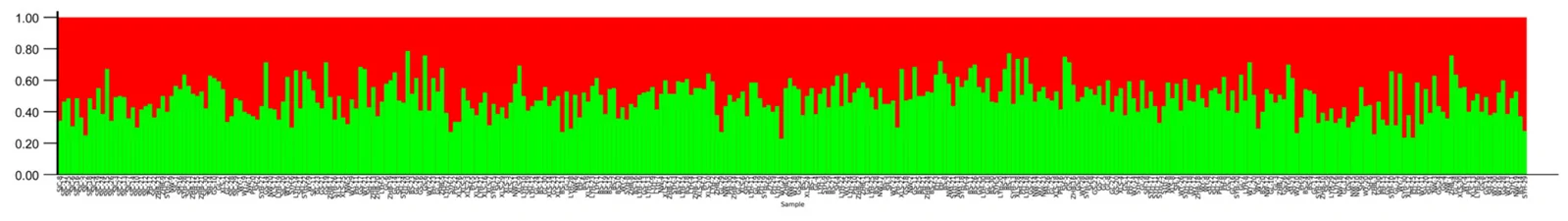
STRUCTURE plot for K = 2. All individuals show admixed ancestry. Vertical bars indicate population boundaries; individual labels on the x-axis indicate population origins.

**Figure 4:**
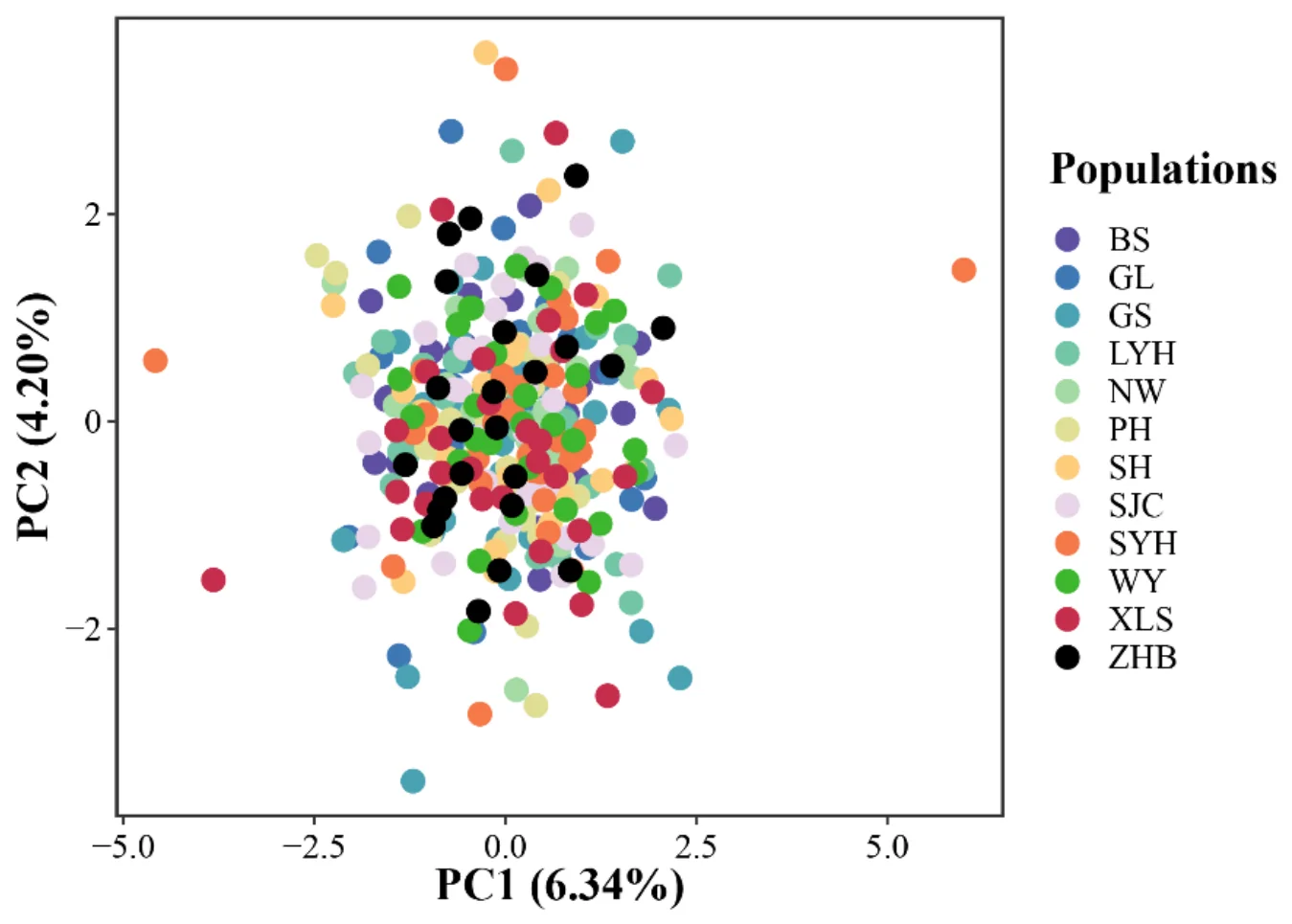
Principal component analysis (PCA) of 342 *M. nipponense* individuals based on 10 polymorphic SSR markers.

To determine the population structure of the total 342 *M. nipponense* individuals using 10 highly polymorphic SSR loci, the STRUCTURE v2.3.4 software was used to infer the number of clusters (K value). When the highest ΔK value was observed, the optimal K value was 2 (Figure 2). However, examination of the posterior probability LnP(K) across K = 1–15 (Supplementary Figure S1; Supplementary Table S2) revealed that the highest LnP(K) value occurred at K = 1 (−14,481.51), with a monotonic decrease thereafter (K = 2 (−14,563.29); K = 3 (−15,122.29)). Moreover, the standard deviation of LnP(K) at K = 2 (26.04) was substantially larger than that at K = 1 (0.27), indicating greater uncertainty in the K = 2 estimate. Following the original approach of Pritchard et al. (2000) [33], which favors the K value with the highest LnP(K) or a clear plateau, K = 1 cannot be confidently rejected as a possible scenario. The ΔK peak at K = 2 should therefore be interpreted with caution, as the Evanno method is known to favour K = 2 even when population structure is weak or absent [36]. The STRUCTURE plot for K = 2 (Figure 3) showed that the 12 populations were divided into two main clusters: one comprising all Huaihe River populations (BS, GL, GS, LYH, NW, PH, SH, SYH, WY, XLS, ZHB) and the other consisting exclusively of the SJC population (Yangtze River outgroup). Extensive admixture was observed among individuals within the Huaihe River cluster, consistent with high gene flow and weak differentiation. PCA showed that the first two principal components explained only 6.34% and 4.20% of total variation (Figure 4), with no clear geographic clustering among the Huaihe River populations, further supporting the absence of strong population structure within the basin. To further evaluate the robustness of the clustering signal, STRUCTURE plots for K = 5 and K = 10 were also examined (Supplementary Figure S2). At higher K values, individuals from all populations showed uniform admixture across clusters without clear differentiation, indicating that the inferred structure at K = 2 likely reflects the presence of the outgroup population (SJC) rather than meaningful biological subdivision within the Huaihe River basin.

### 3.5. Sensitivity Analysis Excluding Mni076

To evaluate whether the inclusion of locus Mni076 biased the main conclusions, we recalculated pairwise *Fst* and *Nm* after excluding this locus and repeated the STRUCTURE analysis with the remaining nine loci. The recalculated *Fst* values ranged from 0.006 to 0.061 (mean = 0.025), and *Nm* values ranged from 3.848 to 41.417 (mean = 14.108), remaining highly similar to those obtained with all ten loci (*Fst*: 0.008–0.058, mean = 0.020; *Nm*: 4.060–31.000). The optimal K value remained K = 2 with the same cluster separation pattern. These results confirm that while Mni076 shows clear evidence of null alleles, its inclusion does not materially affect the key estimates of population differentiation or structure.

## 4. Discussion

The genetic diversity of a population is fundamental to its adaptability, long-term survival, and evolutionary potential [37]. In aquaculture species, maintaining high genetic diversity is essential for sustainable breeding programs and for avoiding inbreeding depression [38,39]. In the present study, the genetic diversity and population structure of 12 *M. nipponense* populations were assessed using ten highly polymorphic microsatellite (SSR) markers. The results provide a comprehensive overview of the genetic status of this species. The ten SSR markers used in this study exhibited high levels of polymorphism. *Na* value is comparable to or even higher than previous reports in *M. nipponense* using microsatellites [10,23]. The mean expected heterozygosity (*He* = 0.805) and polymorphic information content (*PIC* = 0.787) both exceeded the threshold of 0.5, indicating that these markers are highly informative for population genetic analysis [40,41,42]. Notably, the WXM06 locus showed the highest polymorphism (*He* = 0.925, *PIC* = 0.920), while Mni093 displayed relatively lower but still moderate polymorphism (*He* = 0.581, *PIC* = 0.532). These markers validate their utility for future studies on germplasm identification, molecular breeding, and conservation genetics of *M. nipponense*.

At the population level, all 12 populations maintained relatively high genetic diversity, which is consistent with the results of other geographical populations [43]. These studies indicate that the genetic resources of *M. nipponense* are abundant in the Huaihe River and have good potential for genetic improvement and breeding. In addition, *Ae* and *He* values are generally higher than those reported for some other freshwater shrimp populations in China, such as those from the Yellow River (*He* = 0.46–0.75) [44] and from the Yangtze River (*He* = 0.36–0.79) [45]. The relatively high diversity in the Huaihe River populations may be attributed to the well-connected river system, which facilitates gene flow, and to the absence of strong anthropogenic barriers that would otherwise fragment populations. Among the 12 populations, the GS and SYH populations exhibited the highest *Ae* and *He* values, suggesting that these populations harbor the richest genetic variation and can serve as priority sources for core breeding stocks. In contrast, the XLS population, being a cultured stock, showed the lowest genetic diversity. This is commonly observed in hatchery-reared populations due to founder effects, inbreeding, and artificial selection [46]. Although we did not estimate effective population size for this population, the reduced genetic diversity is consistent with expectations for a cultured stock.

Despite the overall high genetic diversity, we observed a consistent pattern of *Ho* being lower than He across all populations, with a mean *Fis* of 0.070. This heterozygote deficiency has also been reported in other *M. nipponense* populations [47,48] and is common in many aquatic species such as *Portunus trituberculatus* [49] and *Litopenaeus vannamei* [50]. Several factors could explain the observed heterozygote deficiency (*Ho* < *He*). First, null alleles are a likely technical cause, as evidenced by the population-locus level analysis (Supplementary Table S1). Locus Mni076 showed the most striking pattern: it exhibited significant HWE deviations (after FDR correction) and positive *Fis* values in 11 out of 12 populations (91.7%). This locus-specific, cross-population consistency strongly implicates a PCR amplification artefact (e.g., a mutation in the primer binding site) rather than a biological phenomenon such as inbreeding or selection [39]. Similarly, locus Mni082 showed moderate *Fna* (0.099–0.208) and significant *HWE* deviations in four populations (Wuyue Reservoir (WY), GS, Laoya River (LYH), SYH), suggesting a possible combination of null alleles and population-specific factors. In contrast, loci WXM02 and WXM33 showed no or negligible null allele evidence (*Fna* ≤ 0.089) and did not deviate from *HWE* in most populations after correction, indicating that these markers are well-behaved for this species.

Second, inbreeding (*Fis* > 0) could contribute, but the overall mean *Fis* (0.071) is low. After FDR correction, only 20% (24 out of 120) of population-locus pairs showed significantly positive *Fis*. Crucially, 11 of these 24 pairs were from locus Mni076 alone. Excluding Mni076, only 13 out of 108 remaining pairs (12.04%) showed significant positive *Fis*. This indicates that the slight excess of homozygosity is more parsimoniously explained by null alleles at a few problematic loci than by widespread inbreeding across populations. To further evaluate whether the inclusion of locus Mni076 biases our main conclusions, we recalculated pairwise *Fst* and *Nm* after excluding this locus and repeated the STRUCTURE analysis with the remaining nine loci. The *Fst* values (0.006–0.061, mean = 0.025) and *Nm* (3.848–41.417, mean = 14.108) estimates remained highly similar to those obtained with all ten loci, and the optimal K value remained K = 2 with the same cluster separation pattern. Thus, while Mni076 shows clear evidence of null alleles, its inclusion does not materially affect the key estimates of population differentiation or structure. We therefore retained it for completeness and transparency. This indicates that the slight excess of homozygosity is more parsimoniously explained by null alleles at a few problematic loci than by widespread inbreeding across populations. Third, the Wahlund effect (mixing of genetically differentiated subpopulations) is unlikely given the weak overall differentiation (*Fst* = 0.02, AMOVA = 98% within variation) [51]. Fourth, natural selection against heterozygotes is improbable for neutral microsatellite markers [8]. Therefore, while null alleles—particularly at locus Mni076—explain a large part of the observed heterozygote deficiency, we cannot fully exclude minor contributions from population-specific inbreeding (e.g., locus Mni082 in LYH and SYH, or locus Mni114 in GS and ZHB). The inclusion of a negative control in each PCR run and stringent peak-calling criteria increase confidence that these results are not due to contamination or scoring errors.

Given the presence of null alleles at several loci, we cannot unequivocally attribute the heterozygote deficiency to biological causes such as inbreeding or genetic drift. While habitat fragmentation and overfishing may reduce effective population size and potentially lead to inbreeding in wild populations [52], our data do not directly support these inferences without further analyses. Because we did not estimate contemporary effective population size (*Ne*) or test for isolation by distance (IBD), we cannot draw any conclusions about the role of genetic drift, demographic decline, or habitat fragmentation in shaping the observed heterozygote deficiency. Therefore, we recommend that future studies incorporate bottleneck detection software (e.g., BOTTLENECK) and null allele-corrected estimators for *Fst* and genetic distances to refine these interpretations. For the purpose of this study, the heterozygote deficiency serves as an early warning signal that warrants careful monitoring, but it should not be overinterpreted as definitive evidence of inbreeding or genetic drift.

Understanding the genetic structure among populations is crucial for devising effective management and conservation strategies [53]. In this study, AMOVA results showed that only 2% of genetic variation was distributed among populations, while 98% resided within populations, with a low but statistically significant overall Fst value (0.020, *p* < 0.01; Table 7). Pairwise *Fst* analysis further revealed that only 18 out of 66 comparisons (27.3%) showed significant differentiation after FDR correction. Notably, 10 of these 18 significant comparisons involved the SJC population (Yangtze River outgroup), indicating that most of the detectable genetic differentiation is driven by the outgroup rather than by structure within the Huaihe River basin. Among the Huaihe River populations, only 8 out of 55 pairwise comparisons (14.5%) showed significant but very weak differentiation (*Fst* = 0.011–0.026). In areas with high hydrological connectivity, physical barriers are rarely present, allowing for the long-distance dispersal of both juveniles and adults. This facilitates extensive gene flow, which in turn leads to low *Fst* and high *Nm* values—a pattern commonly observed in freshwater species continuously distributed across large, interconnected river basins. However, it is important to note that *Nm* is directly derived from *Fst* and is not an independent estimate of contemporary migration. The low differentiation observed may therefore reflect a combination of ongoing gene flow, historical connectivity, recent common ancestry, or a combination of these factors. Consequently, the Huaihe River populations can be considered a single genetic management unit for conservation and breeding purposes. However, we acknowledge that biologically meaningful conservation units cannot be defined from neutral microsatellite data alone, particularly with only ten markers. Future studies incorporating adaptive loci, phenotypic data, and more comprehensive geographic sampling are needed to refine conservation unit designations.

Despite the overall homogeneity indicated by low *Fst* and high gene flow, STRUCTURE analysis (K = 2) detected a subtle clustering signal, separating the Yangtze River outgroup (SJC) from all Huaihe River populations. However, evaluation of the posterior probability LnP(K) following Pritchard et al. (2000) [33] revealed that the highest LnP(K) value occurred at K = 1 (−14,481.51), with values decreasing monotonically thereafter (K = 2 (−14,563.29); K = 3 (−15,122.29)) (Supplementary Figure S1). Moreover, the standard deviation at K = 2 (26.04) was substantially larger than at K = 1 (0.27), indicating greater uncertainty in the K = 2 estimate. The ΔK method is known to favour K = 2 even when population structure is weak or absent [36], and the lack of a clear plateau in LnP(K) further supports the conclusion that K = 1 cannot be confidently rejected as a possible scenario. Therefore, while the ΔK peak at K = 2 is statistically detectable, it is likely a methodological artefact reflecting the presence of the SJC outgroup rather than a meaningful biological subdivision within the Huaihe River basin. It is crucial to distinguish between statistical significance and biological significance: although the STRUCTURE signal and pairwise *Fst* values were statistically significant (*p* < 0.05), the extremely low Fst (0.008–0.058) and 98% within-population variation indicate that this differentiation is biologically negligible. Such weak signals can arise from highly polymorphic SSR markers even under extensive gene flow, and the main differentiation was largely driven by the SJC outgroup. Indeed, one of the two clusters consisted almost exclusively of individuals from the SJC population (Yangtze River outgroup), indicating that the detected subdivision primarily reflects inter-basin differentiation rather than any meaningful genetic structure within the Huaihe River basin. This subdivision was not strictly geographical, as individuals from the same sampling site often exhibited mixed ancestry proportions (Figure 3). The PCA plot further supported this pattern: the first two principal components explained only 6.34% and 4.2% of the total variation, respectively, and no clear geographic clustering was observed, denoting that genetic differentiation among populations is weak and not structured by spatial distance (Figure 4).

Our findings are broadly consistent with previous genetic studies conducted in the Huaihe River basin. Cui et al. (2018) [11] used mitochondrial D-loop sequences to analyze *M. nipponense* populations from the Huaihe River and reported high haplotype diversity (*Hd* = 0.987) and low genetic differentiation, which aligns with our observation of high genetic diversity and weak population structure. Similarly, Jiang et al. (2012) [24] used nine microsatellite markers to analyze populations from the Anhui section of the Huaihe River and found moderate to high genetic diversity (*He* = 0.632–0.732) and low differentiation (*Fst* = 0.018–0.063), consistent with our results. However, some differences exist: Cui et al. (2018) [11] identified a weak phylogeographic break between upper and lower Huaihe River populations based on mitochondrial haplotypes, whereas our SSR data did not reveal such geographic structuring. This discrepancy may reflect differences in marker type (mitochondrial vs. nuclear) and geographic coverage. Mitochondrial markers are maternally inherited and more sensitive to historical demographic events [12], while nuclear SSRs provide a genome-wide perspective and are better suited for detecting contemporary gene flow [13]. Additionally, our sampling was concentrated in the upper Huaihe River basin, whereas Cui et al. (2018) [11] included populations from both upper and lower reaches. The absence of geographic structure in our study may also reflect extensive gene flow within the upper basin, which could erase signals of historical phylogeographic breaks [16,17,18]. Overall, despite minor discrepancies, the preponderance of evidence from both mitochondrial and nuclear markers supports the conclusion that *M. nipponense* in the Huaihe River maintains high genetic diversity and weak population differentiation.

Collectively, these results illuminate that historical rather than contemporary barriers have shaped the genetic architecture. For instance, Cui et al. (2018) [11] estimated that during the early Late Pleistocene, M. nipponense populations in the Huaihe River experienced a population expansion approximately 85,500 years ago. This period was characterised by repeated glacial-interglacial cycles, which likely caused habitat fragmentation and temporary isolation of distinct groups. Subsequent post-glacial climate warming and re-connection of river systems would have promoted secondary contact and extensive gene flow, erasing most of the initial differentiation but leaving a subtle, geographically non-specific genetic structure (e.g., the scattered PCA pattern). Thus, the weak population subdivision observed in our study may represent a ‘residual signature’ of Pleistocene isolation followed by recent admixture, rather than ongoing barriers to dispersal. Therefore, the Huaihe River populations can be viewed as functioning as a largely connected metapopulation system, where high gene flow and hydrological connectivity override any incipient structuring.

The findings of this study have direct implications for the management and sustainable utilization of *M. nipponense* germplasm resources in the Huaihe River. However, given the weak overall genetic differentiation observed (low *Fst*, high gene flow, and extensive admixture) and the use of only ten neutral SSR markers, we suggest: (1) periodic monitoring of genetic diversity using the validated SSR markers to track potential changes over time; (2) given the overall homogeneity, intensive selection within already high-diversity populations (e.g., GS, SYH) is a reasonable strategy for breeding programs; (3) establishing a germplasm bank covering all populations to protect genetic resources from potential environmental disasters or overfishing.

Moreover, several limitations of this study should be acknowledged. The number of SSR markers (ten) is relatively small, and the markers are neutral, providing no information on adaptive variation. Therefore, we cannot infer local adaptation or define biologically meaningful conservation units. Future studies should increase marker density using whole-genome resequencing or SNP arrays and, if possible, incorporate phenotypic data to assess adaptive traits. We also plan to develop and incorporate an additional panel of SSR markers in our ongoing research to improve the resolution of population genetic analyses. Additionally, our sampling was limited to the upper Huaihe River basin; comparisons with populations from other river systems (e.g., the Yangtze, Yellow, and Pearl Rivers) would help to understand the broader phylogeographic pattern of *M. nipponense*. The inclusion of SJC as an outgroup from the Yangtze River provides only preliminary evidence of inter-basin differentiation, and more comprehensive sampling across multiple river systems is needed. Finally, we did not investigate associations between genetic diversity and adaptive traits (e.g., growth rate, disease resistance); integrating genomic and phenotypic data remains a promising direction for marker-assisted breeding but is beyond the scope of this preliminary study.

## 5. Conclusions

In summary, this study reveals that *M. nipponense* populations in the Huaihe River maintain high genetic diversity, with GS and SYH showing the highest variation. A consistent heterozygote deficiency was observed, which is more parsimoniously explained by null alleles at specific loci (e.g., Mni076) than by widespread inbreeding. Genetic differentiation among populations is weak, and the subtle STRUCTURE clustering signal (K = 2) likely reflects the presence of the SJC outgroup rather than meaningful subdivision within the Huaihe basin, as LnP(K) did not confidently reject K = 1. While these results provide a scientific basis for prioritizing high-diversity populations for breeding and genetic monitoring, we emphasize that ten neutral SSR markers are insufficient to fully characterize adaptive variation or definitively define conservation units. Conclusions regarding gene flow and marker-assisted selection should therefore be treated with caution and require further validation through expanded genomic markers, phenotypic evaluations, and broader geographic sampling.

## Figures and Tables

**Table 1:** Sample information of twelve *M. nipponense* populations.

Sampling Site	Source	Longitude (E°)	Latitude (N°)	Sample Size
Boshan Reservoir (BS)	Huaihe River	113°52′30″	32°42′48″	30
Huai River mainstream (GL)		115°53′22″	32°34′42″	30
Guangshan Nature Reserve (GS)		114°52′37″	31°46′56″	30
Laoya River (LYH)		113°51′52″	32°27′5″	30
Nanwan Reservoir (NW)		113°59′57″	32°7′28″	23
Pohe Reservoir (PH)		114°59′13″	32°7′28″	30
Shiguan River (SH)		115°17′15″	31°28′9″	25
Suyahu Reservoir (SYH)		114°10′44″	33°5′47″	30
Wuyue Reservoir (WY)		114°38′29″	31°52′15″	30
Xiaolong Mountain (XLS)		114°31′12″	32°10′48″	30
Chinese Turtle Reserve Area (ZHB)		115°6′36″	32°18′10″	24
Songjiachang Reservoir (SJC)	Yangtze River	113°29′48″	32°45′50″	30

**Table 2:** Sequence of 10 pairs of microsatellite primers.

Loci	Primer Sequence	Repeat Motif	Tm (°C)	References
WXM02	F: GCCATTTTCTCATAAGGGT	(AG)19	54	[21]
	R: ACGGTGGTATTCAGGGAT			
WXM06	F: TTGGCAAGTCTCGTCTGATG	(GA)7(AG)19	54	
	R: CGAGGAAACGCCTGCTAC			
Mni114	F: CTCGTTCTTGCCACTCTT	(TG)22(GA)29	50	
	R: TGACCCGTCTATGTTCGT			
Mni06	F: GGCTATAAAGCGACCCTGAAT	(AC)11	50	[22]
	R: CCCAGCCCTGTGATATTTTTAC			
Mni013	F: TAGATGCAGCAGTAAAGCAAATGA	(GA)20	50	
	R: TTCACACTTCCCCATCAGTATCTC			
Mni076	F: GTTCTCAGCCTTCTCCAT	(AG)10…(AG)11	55	
	R: GTTTATCGCTTTATTAGTCC			
Mni082	F: AACAAAGGTGAAATGCCACA	(GT)7…(TG)23	54	[23]
	R: CCGCCAAATCAACAGTAATC			
Mni093	F: TCACAGCACGAGATAACA	(TG)4…(TG)15	53	[24]
	R: TGCCGATTCTGGACTTTA			
Mni30	F: TGCTGTTATCGTTGGCTGAG	(AC)11	55	
	R: ACCCGTGTGTGTGAACTGAG			
WXM33	F: TGGTCCTGTTGTCTTTCT	(CT)18	54	[25]
	R: GTTGCTCGGTCTATGC			

F: Forward primer; R: Reverse primer.

**Table 3:** Genetic parameters for 10 SSR loci.

Loci	*Na*	*Ae*	*I*	*Ho*	*He*	*PIC*
Mni30	22	5.769	2.038	0.790	0.827	0.805
Mni06	11	3.410	1.472	0.689	0.707	0.667
Mni013	22	12.796	2.692	0.900	0.922	0.916
Mni076	17	3.432	1.772	0.362	0.709	0.688
Mni082	23	7.971	2.348	0.738	0.875	0.863
Mni093	13	2.384	1.224	0.518	0.581	0.532
Mni114	27	10.141	2.61	0.834	0.901	0.894
WXM02	32	3.695	2.078	0.678	0.729	0.717
WXM06	29	13.354	2.810	0.902	0.925	0.92
WXM33	17	8.025	2.251	0.830	0.875	0.863
Mean	21.300	7.098	2.130	0.724	0.805	0.787

*Na*: Number of alleles; *Ae*: Number of effective alleles; *I*: Shannon’s diversity index; *Ho*: Observed heterozygosity; *He*: Expected heterozygosity; *PIC*: Polymorphism information content.

**Table 4:** Genetic diversity indices of twelve *M. nipponense* populations.

Populations	*Na*	*Ae*	*I*	*Ho*	*He*	*Fis*	*HWE*
BS	10.400	5.393	1.818	0.712	0.761	−0.003	*
GL	13.000	6.358	2.004	0.689	0.788	0.072	***
GS	12.900	6.699	2.036	0.697	0.793	0.062	***
LYH	10.100	5.443	1.827	0.729	0.769	−0.056	**
NW	10.700	5.871	1.905	0.738	0.781	−0.108	***
PH	11.100	6.072	1.942	0.763	0.796	−0.002	**
SH	11.700	5.952	1.932	0.737	0.771	−0.009	**
SJC	8.500	4.968	1.695	0.713	0.760	0.221	*
SYH	13.000	6.663	2.055	0.767	0.803	−0.016	*
WY	11.100	5.606	1.864	0.712	0.754	0.005	*
XLS	8.900	4.783	1.694	0.695	0.742	0.057	**
ZHB	11.100	6.134	1.883	0.741	0.759	−0.019	**
Mean	11.040	5.829	1.888	0.724	0.773	0.017	

*Na*: Number of alleles; *Ae*: Number of effective alleles; *I*: Shannon’s diversity index; *Ho*: Observed heterozygosity; *He*: Expected heterozygosity; *Fis*: inbreeding coefficient; *HWE*: Hardy–Weinberg Equilibrium; * *p* < 0.05; ** *p* < 0.01; *** *p* < 0.001.

**Table 5:** Genetic differentiation coefficient (*Fst*, below diagonal) and gene flow (*Nm*, above diagonal) among twelve *M. nipponense* populations.

Populations	BS	GL	GS	LYH	NW	PH	SH	SJC	SYH	WY	XLS	ZHB
BS	-	15.375	11.655	11.114	13.639	12.908	12.250	4.295	12.908	9.365	12.250	11.114
GL	0.016	-	27.528	15.375	20.583	17.607	31.000	4.852	17.607	16.417	16.417	22.477
GS	0.021 *	0.009	-	15.375	22.477	18.981	17.607	5.185	17.607	14.456	13.639	17.607
LYH	0.022	0.016 **	0.016	-	14.456	12.250	12.908	4.060	11.655	11.655	11.114	11.655
NW	0.018	0.012 **	0.011 *	0.017	-	22.477	17.607	5.702	15.375	12.908	17.607	17.607
PH	0.019 *	0.014	0.013 *	0.020	0.011	-	14.456	6.160	18.981	10.620	17.607	13.639
SH	0.020	0.008	0.014	0.019	0.014	0.017 *	-	4.558	20.583	17.607	14.456	24.750
SJC	0.055 **	0.049	0.046 *	0.058 **	0.042	0.039	0.052 **	-	6.329	4.214	4.652	4.558
SYH	0.019	0.014	0.014	0.021	0.016 **	0.013	0.012	0.038 **	-	12.250	14.456	18.981
WY	0.026	0.015 **	0.017	0.021 **	0.019 *	0.023	0.014	0.056	0.020	-	10.167	15.375
XLS	0.020	0.015 *	0.018	0.022	0.014	0.014 **	0.017	0.051 *	0.017 *	0.024	-	14.456
ZHB	0.022 **	0.011	0.014	0.021 *	0.014	0.018 **	0.010	0.052 *	0.013	0.016 **	0.017	-

* *p* < 0.05; ** *p* < 0.01 (after FDR correction).

**Table 6:** Genetic distance among twelve *M. nipponense* populations.

Populations	BS	GL	GS	LYH	NW	PH	SH	SJC	SYH	WY	XLS	ZHB
BS	-											
GL	0.145	-										
GS	0.178	0.120	-									
LYH	0.141	0.138	0.133	-								
NW	0.167	0.135	0.118	0.126	-							
PH	0.153	0.121	0.115	0.121	0.094	-						
SH	0.176	0.098	0.134	0.141	0.133	0.145	-					
SJC	0.277	0.253	0.252	0.258	0.216	0.215	0.234	-				
SYH	0.137	0.121	0.119	0.113	0.138	0.122	0.129	0.201	-			
WY	0.186	0.145	0.151	0.153	0.158	0.162	0.138	0.265	0.163	-		
XLS	0.165	0.139	0.159	0.130	0.129	0.129	0.143	0.246	0.144	0.179	-	
ZHB	0.169	0.120	0.128	0.138	0.126	0.127	0.131	0.216	0.152	0.159	0.149	-

**Table 7:** Analysis of molecular variance (AMOVA) from twelve *M. nipponense* populations.

Source of Variation	*df*	*SS*	Variance Component	Percentage of Variation	F-Statistics
Among populations	11	107.299	0.093	2%	**
Within populations	672	2678.342	3.993	98%	
Total	683	2785.640	4.087	100%	

*df*: Degrees of freedom; *SS*: Sum of squares; ** *p* < 0.01 (based on 10,000 permutations).

## Data Availability

The original contributions presented in this study are included in the article. Further inquiries can be directed to the corresponding author.

## Supplementary Materials

The following supporting information can be downloaded at: https://www.mdpi.com/article/10.3390/genes17080880/s1, Figure S1: Mean LnP(K) values (±SD) across K = 1–15 from STRUCTURE analysis. The highest LnP(K) value occurred at K = 1, with values decreasing monotonically thereafter; Figure S2: STRUCTURE plot for K = 2 and K = 5. All individuals show admixed ancestry. Vertical bars indicate population boundaries; population codes are indicated below the x-axis; Table S1: Genetic diversity parameters of 12 *M. nipponense* populations; Table S2: Summary of STRUCTURE analysis results for K = 1–15, including mean LnP(K), standard deviation, and ΔK values.
