# Supplementary material for: Genetic Diversity and Population Structure of Macrobrachium nipponense in Huaihe River Using Microsatellite Markers
Source: Genes (Basel). 2026 Jul 28;17(8):880. doi: 10.3390/genes17080880 (PMC13511783; doi:10.3390/genes17080880)
Supplement: Supplementary file 1 [file genes-17-00880-s001.zip › genes-4442047-supplementary.pdf]

Supplementary Table S1. Genetic diversity parameters of 12 *M. nipponense* populations.

**Population**	**Locus**	***Na***	***Ae***	***I***	***Ho***	***He***	***Fis***	***Fna***	***HWE***
BS	Mni30	10.000	5.289	1.879	0.828	0.811	-0.021	0.000	ns
	Mni06	7.000	3.168	1.397	0.621	0.684	0.093	0.017	ns
	Mni013	13.000	8.867	2.327	0.900	0.887	-0.014	0.002	ns
	Mni076	8.000	2.832	1.360	0.207	0.647	0.680	0.261	***
	Mni082	12.000	6.475	2.082	0.733	0.846	0.133	0.054	ns
	Mni093	5.000	2.079	1.000	0.448	0.519	0.136	0.061	ns
	Mni114	18.000	8.696	2.487	0.967	0.885	-0.092	0.000	ns
	WXM02	10.000	2.717	1.464	0.690	0.632	-0.091	0.000	ns
	WXM06	13.000	8.452	2.309	0.862	0.882	0.022	0.000	ns
	WXM33	8.000	5.357	1.871	0.867	0.813	-0.066	0.000	ns
GL	Mni30	10.000	5.051	1.858	0.759	0.802	0.054	0.020	ns
	Mni06	7.000	2.941	1.368	0.700	0.660	-0.061	0.005	ns
	Mni013	17.000	10.909	2.574	0.900	0.908	0.009	0.019	ns
	Mni076	11.000	3.532	1.656	0.286	0.717	0.601	0.242	***
	Mni082	11.000	7.127	2.124	0.690	0.860	0.198	0.097	ns
	Mni093	8.000	2.387	1.276	0.433	0.581	0.254	0.094	**
	Mni114	15.000	9.045	2.410	0.833	0.889	0.063	0.025	ns
	WXM02	16.000	3.041	1.770	0.567	0.671	0.156	0.027	ns
	WXM06	19.000	11.688	2.672	0.967	0.914	-0.057	0.000	ns
	WXM33	16.000	7.860	2.330	0.759	0.873	0.131	0.067	**
GS	Mni30	13.000	6.164	2.130	0.800	0.838	0.045	0.068	ns
	Mni06	7.000	2.998	1.402	0.643	0.666	0.035	0.000	ns
	Mni013	18.000	12.329	2.665	0.867	0.919	0.057	0.039	***
	Mni076	9.000	4.521	1.783	0.435	0.779	0.442	0.217	**
	Mni082	14.000	8.411	2.341	0.800	0.881	0.092	0.108	*
	Mni093	5.000	2.250	0.996	0.464	0.555	0.164	0.033	ns
	Mni114	19.000	11.321	2.664	0.733	0.912	0.196	0.118	***
	WXM02	13.000	2.753	1.639	0.448	0.637	0.296	0.089	***
	WXM06	20.000	9.679	2.646	0.929	0.897	-0.036	0.000	ns
	WXM33	11.000	6.568	2.094	0.852	0.848	-0.005	0.017	ns
LYH	Mni30	9.000	5.202	1.842	0.867	0.808	-0.073	0.000	ns
	Mni06	7.000	3.261	1.399	0.700	0.693	-0.010	0.036	ns
	Mni013	13.000	7.200	2.192	0.867	0.861	-0.006	0.000	ns
	Mni076	9.000	4.534	1.761	0.567	0.779	0.273	0.121	**
	Mni082	9.000	5.070	1.841	0.433	0.803	0.460	0.208	***
	Mni093	7.000	2.353	1.202	0.633	0.575	-0.101	0.000	ns
	Mni114	13.000	7.067	2.179	0.793	0.859	0.076	0.014	ns
	WXM02	11.000	2.273	1.385	0.533	0.560	0.048	0.000	ns
	WXM06	15.000	11.111	2.525	0.967	0.910	-0.062	0.000	ns
	WXM33	8.000	6.360	1.946	0.933	0.843	-0.107	0.000	ns
NW	Mni30	8.000	5.095	1.794	0.909	0.804	-0.131	0.000	ns
	Mni06	4.000	2.792	1.153	0.609	0.642	0.052	0.015	ns

	Mni013	13.000	10.173	2.420	1.000	0.902	-0.109	0.000	ns
	Mni076	10.000	4.438	1.856	0.389	0.775	0.498	0.212	**
	Mni082	14.000	7.149	2.267	0.826	0.860	0.040	0.057	ns
	Mni093	5.000	2.246	1.050	0.391	0.555	0.295	0.134	***
	Mni114	12.000	8.274	2.243	0.864	0.879	0.018	0.025	ns
	WXM02	12.000	3.130	1.725	0.696	0.681	-0.022	0.000	ns
	WXM06	17.000	10.173	2.558	0.913	0.902	-0.013	0.000	ns
	WXM33	12.000	5.238	1.984	0.783	0.809	0.033	0.023	ns
PH	Mni30	9.000	4.651	1.761	0.800	0.785	-0.019	0.009	ns
	Mni06	6.000	3.468	1.461	0.767	0.712	-0.077	0.000	ns
	Mni013	14.000	9.730	2.420	0.967	0.897	-0.077	0.000	ns
	Mni076	9.000	3.303	1.580	0.400	0.697	0.426	0.183	***
	Mni082	11.000	7.229	2.144	0.900	0.862	-0.044	0.022	ns
	Mni093	6.000	2.507	1.103	0.533	0.601	0.113	0.015	ns
	Mni114	15.000	7.895	2.321	0.833	0.873	0.046	0.069	ns
	WXM02	13.000	4.569	1.973	0.767	0.781	0.018	0.027	ns
	WXM06	17.000	10.778	2.593	0.833	0.907	0.081	0.091	ns
	WXM33	11.000	6.593	2.067	0.833	0.848	0.018	0.018	ns
SH	Mni30	14.000	5.435	2.058	0.840	0.816	-0.029	0.000	ns
	Mni06	6.000	3.378	1.427	0.760	0.704	-0.080	0.000	ns
	Mni013	14.000	9.542	2.405	0.880	0.895	0.017	0.025	ns
	Mni076	9.000	2.432	1.318	0.261	0.589	0.557	0.208	***
	Mni082	12.000	7.245	2.188	0.792	0.862	0.082	0.063	ns
	Mni093	7.000	2.264	1.217	0.600	0.558	-0.074	0.000	ns
	Mni114	11.000	7.062	2.121	0.880	0.858	-0.025	0.003	ns
	WXM02	14.000	2.828	1.706	0.640	0.646	0.010	0.000	ns
	WXM06	18.000	11.468	2.661	0.880	0.913	0.036	0.026	ns
	WXM33	12.000	7.862	2.220	0.840	0.873	0.038	0.031	ns
SJC	Mni30	5.000	2.699	1.193	0.500	0.629	0.206	0.075	ns
	Mni06	4.000	3.292	1.243	0.621	0.696	0.108	0.037	ns
	Mni013	13.000	7.347	2.239	0.933	0.864	-0.080	0.000	ns
	Mni076	4.000	2.399	0.993	0.160	0.583	0.726	0.272	***
	Mni082	7.000	4.991	1.692	0.724	0.800	0.094	0.040	ns
	Mni093	7.000	3.046	1.387	0.733	0.672	-0.092	0.000	ns
	Mni114	12.000	6.184	2.084	0.897	0.838	-0.070	0.009	ns
	WXM02	10.000	4.959	1.831	0.867	0.798	-0.086	0.000	ns
	WXM06	14.000	8.491	2.312	0.833	0.882	0.055	0.008	ns
	WXM33	9.000	6.272	1.975	0.867	0.841	-0.031	0.000	ns
SYH	Mni30	13.000	6.029	2.068	0.862	0.834	-0.033	0.000	ns
	Mni06	9.000	4.072	1.636	0.800	0.754	-0.060	0.000	ns
	Mni013	16.000	10.843	2.520	0.867	0.908	0.045	0.009	ns
	Mni076	9.000	2.122	1.255	0.346	0.529	0.345	0.071	*
	Mni082	13.000	8.333	2.277	0.667	0.880	0.242	0.117	***
	Mni093	8.000	2.782	1.342	0.533	0.641	0.167	0.095	ns

	Mni114	18.000	8.955	2.499	0.867	0.888	0.024	0.000	ns
	WXM02	17.000	5.825	2.279	0.833	0.828	-0.006	0.000	ns
	WXM06	14.000	10.526	2.486	0.967	0.905	-0.068	0.000	ns
	WXM33	13.000	7.143	2.187	0.933	0.860	-0.085	0.000	ns
WY	Mni30	8.000	3.986	1.617	0.759	0.749	-0.013	0.000	ns
	Mni06	6.000	2.635	1.226	0.600	0.621	0.033	0.051	*
	Mni013	13.000	8.295	2.280	0.833	0.879	0.052	0.009	ns
	Mni076	9.000	3.613	1.689	0.643	0.723	0.111	0.050	ns
	Mni082	11.000	7.345	2.177	0.690	0.864	0.202	0.099	**
	Mni093	5.000	1.557	0.695	0.333	0.358	0.068	0.003	ns
	Mni114	12.000	6.979	2.182	0.862	0.857	-0.006	0.000	ns
	WXM02	18.000	3.947	2.094	0.733	0.747	0.018	0.000	ns
	WXM06	19.000	11.842	2.697	0.867	0.916	0.053	0.064	ns
	WXM33	10.000	5.863	1.982	0.800	0.829	0.035	0.000	ns
XLS	Mni30	8.000	4.545	1.657	0.750	0.780	0.038	0.000	ns
	Mni06	5.000	3.161	1.319	0.786	0.684	-0.149	0.000	ns
	Mni013	14.000	7.840	2.316	0.857	0.872	0.018	0.016	ns
	Mni076	8.000	2.307	1.226	0.310	0.567	0.452	0.178	***
	Mni082	8.000	4.467	1.697	0.733	0.776	0.055	0.000	ns
	Mni093	4.000	2.195	0.962	0.567	0.544	-0.041	0.000	ns
	Mni114	11.000	6.316	2.039	0.767	0.842	0.089	0.021	ns
	WXM02	9.000	3.121	1.588	0.655	0.680	0.036	0.000	ns
	WXM06	15.000	9.116	2.425	0.857	0.890	0.037	0.000	ns
	WXM33	7.000	4.762	1.708	0.667	0.790	0.156	0.053	ns
ZHB	Mni30	11.000	6.080	2.041	0.870	0.836	-0.041	0.014	ns
	Mni06	4.000	2.806	1.156	0.636	0.644	0.011	0.000	ns
	Mni013	17.000	11.755	2.618	0.958	0.915	-0.047	0.000	ns
	Mni076	7.000	2.192	1.169	0.300	0.544	0.448	0.159	***
	Mni082	15.000	8.963	2.393	0.955	0.888	-0.074	0.000	ns
	Mni093	6.000	1.843	0.964	0.522	0.457	-0.140	0.000	ns
	Mni114	16.000	9.043	2.454	0.696	0.889	0.218	0.130	***
	WXM02	12.000	3.049	1.598	0.696	0.672	-0.035	0.000	ns
	WXM06	14.000	8.744	2.389	0.957	0.886	-0.080	0.000	ns
	WXM33	9.000	6.865	2.044	0.818	0.854	0.042	0.043	*

Na: Number of alleles; *Ae*: Number of effective alleles; *I*: Shannon's diversity index; *Ho*: Observed heterozygosity; *He*: Expected heterozygosity; *Fis*: inbreeding coefficient; *Fna*: Null allele frequencies; *HWE*: Hardy-Weinberg Equilibrium; *$P < 0.05$; **$P < 0.01$; ***$P < 0.001$; ns: no deviations from *HWE*.

Supplementary Table S2. Summary of STRUCTURE analysis results for K = 1–15, including mean LnP(K), standard deviation, and ΔK values.

K	Mean LnP(K)	Stdev LnP(K)	Ln'(K)	|Ln''(K)|	Delta K
1	-14481.51	0.26854	NA	NA	NA
2	-14563.29	26.04148	-81.78	477.22	18.32538
3	-15122.29	1045.26136	-559	872.17	0.8344
4	-14809.12	194.06588	313.17	107.85	0.55574
5	-14603.8	111.40468	205.32	266.89	2.39568
6	-14665.37	149.90016	-61.57	115.77	0.77231
7	-14842.71	52.60052	-177.34	54.66	1.03915
8	-14965.39	131.33963	-122.68	42.84	0.32618
9	-15045.23	135.23843	-79.84	70.45	0.52093
10	-15195.52	113.30865	-150.29	211.77	1.86897
11	-15557.58	374.03387	-362.06	161.2	0.43098
12	-15758.44	161.89944	-200.86	207.08	1.27907
13	-16166.38	542.14159	-407.94	56.67	0.10453
14	-16517.65	282.86602	-351.27	188.46	0.66625
15	-16680.46	472.14144	-162.81	NA	NA

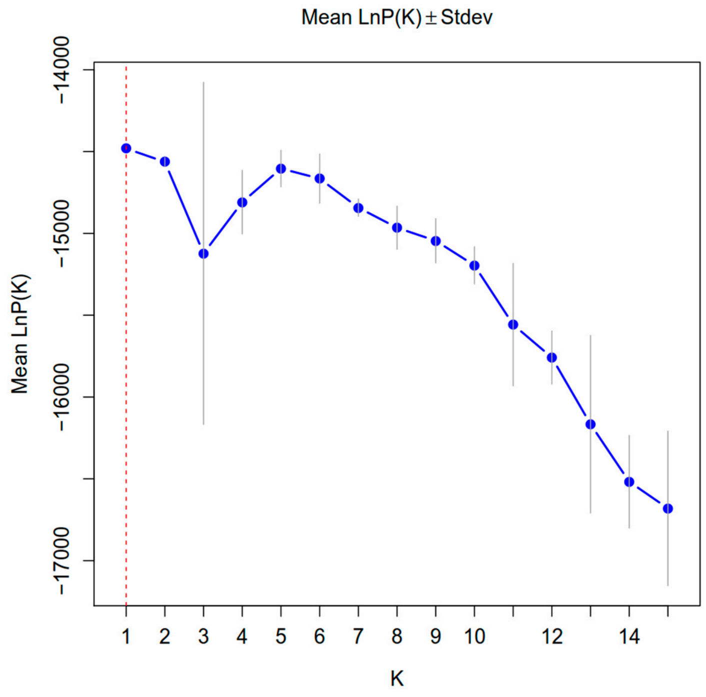

Supplementary Figure S1. Mean LnP(K) values (±SD) across K = 1–15 from STRUCTURE analysis. The highest LnP(K) value occurred at K = 1, with values decreasing monotonically thereafter.

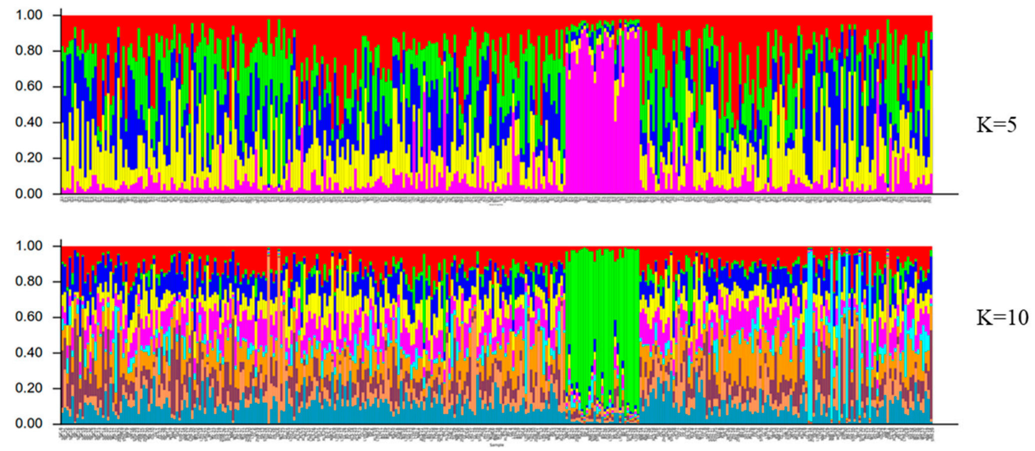

Supplementary Figure S2. STRUCTURE plot for K = 2 and K = 5. All individuals show admixed ancestry. Vertical bars indicate population boundaries; population codes are indicated below the x-axis.
